# Switching to instant black coffee modulates sodium selenite-induced cataract in rats

**DOI:** 10.3205/000232

**Published:** 2016-04-25

**Authors:** E. A. El Okda, M. M. Mohamed, E. B. Shaheed, A. R. Abdel-Moemin

**Affiliations:** 1 Department of Home Economics, Women’s College, Ain Shams University, Cairo, Egypt; 2 Pathology Department, Faculty of Veterinary Medicine, Cairo University, Cairo, Egypt; 3 Department of Nutrition and Food Science, Faculty of Home Economics, Helwan University, Cairo, Egypt

**Keywords:** instant black coffee, black tea, hibiscus, total phenols, sodium selenite, cataract, rats, co-phenols

## Abstract

The influence of daily consumption of some common beverages on the development of cataract in rats was investigated. Total phenol content was determined in the beverages and an oral standardized dose of total phenols from each beverage was given to the treated rats. Weaned male albino rats were used and divided into five groups (n=7). Rats were fed Ain 93G and administered the standardized dose of instant coffee, black tea and hibiscus beverages for 30 days. On day 14 all rats were injected with a single dose of sodium selenite (Na_2_SeO_3_) 15 µmol/kg bodyweight, except the control groups NC (negative control, did not receive Na_2_SeO_3_) and PC (positive control, was already injected on day 1 of the study). The rats were continued on Ain 93G and the standardized dose for another 16 days. Positive control rats were used. Total phenols were 210, 40, and 44 mg/g dry weight gallic acid equivalent in black coffee, black tea, and hibiscus, respectively. Decreased levels (statistically significant P<0.05) of malondialdehyde, total nitric oxide, Ca-ATPase, tumor necrosis factor-α, interleukin-1β, superoxide dismutase, and conversely, increased levels (statistically significant P<0.05) of total protein, reduced glutathione, catalase were found in the lenses of the coffee group compared to PC. There are co-phenol substances in the instant black coffee that promoted coffee to be the most effective beverage.

## Zusammenfassung

Der Einfluss des täglichen Konsums einiger üblicher Getränke auf die Entwicklung einer Katarakt wurde in Ratten untersucht. Der Gesamtphenolgehalt der Getränke wurde bestimmt und eine standardisierte Menge an Gesamtphenol jedes Getränks wurde an die Ratten verfüttert. Abgestillte Albinoratten wurden in 5 Gruppen (n=7) aufgeteilt, die alle mit Ain93G gefüttert wurden. Über 30 Tage erhielten die behandelten Ratten eine festgelegte Standarddosis an Instant-Kaffee, schwarzem Tee bzw. eine Hibiskuszubereitung. Am 14. Tag wurde den Ratten eine Dosis Natriumselenit (15 µmol/kg Körpergewicht) gespritzt, ausgenommen die negative Kontrollgruppe, die kein Selenit bekam, und die positive Kontrollgruppe, die bereits am 1. Tag der Studie die Selenitinjektion erhielt. Die Ratten wurden weitere 16 Tage mit Ain93G gefüttert und erhielten die festgelegte Menge an Kaffee, Tee bzw. Hibiscus. Die Gsamtphenolgehalte von Kaffee, schwarzem Tee bzw. Hibiskus waren jeweils 210, 40 und 44 mg/g Trockengewicht Gallussäure-Äquivalente. In den Linsen der Kaffee-Gruppe wurden im Vergleich zur positiven Kontrollgruppe verminderte Gehalte an Malondialdehyd, Gesamt-Stickstoffoxid, Calcium-ATPase, Tumornekrosefaktor-alpha, Interleukin-1beta und Superoxiddismutase gemessen, andererseits erhöhte Gehalte ( p<0,05) an Gesamtprotein, reduziertem Glutathion und Katalase. Co-Phenol-Substanzen im schwarzen Instant-Kaffee bewirken, dass Kaffee als wirksamstes Nahrungsmittel gegen die Entstehung der Katarakt bei Ratten angesehen werden kann.

## Introduction

Cataract is the loss of eye lens transparency that means cloudiness or opacification of the eye lens. Cataract represents one of the major causes of visual impairment globally and the first cause of blindness, accounting for 51% of cases worldwide [1], [2]. It is also estimated that about 40 million people will have severely reduced vision due to cataract by the year 2020, especially in Asia and Africa [3].

Age-related cataract is the leading cause of world blindness. Until recently, the biochemical mechanisms that result in human cataract formation have remained in obscurity [4]. Smoking, excessive UV-B exposure, diabetes, and steroid treatment are also known as cataract risk factors [5]. 

Both epidemiological and experimental studies provide evidence that oxidative stress is a major mechanism in the initiation and progression of cataracts [6], [7], [8]. Accordingly, lenses have evolved antioxidant systems to defend against the toxic damage of reactive oxygen species (ROS), including antioxidants, such as reduced glutathione (GSH), superoxide dismutase (SOD), catalase (CAT), glutathione S-transferase (GST), and glutathione reductase/peroxidase (GR/GPX) [9].

Natural antioxidants molecules have been reported to be an indispensable application in prevention and control of cataract due to their easy availability and less complications [10], [11]. Quercetin was found to inhibit hydrogen peroxide induced cataract [12]. Isorhamnetin-3-glucoside found its application on selenite-induced cataract [13] and catechins derivatives were very efficient in inhibiting N-methyl-N-nitrosourea-induced cataract in rats [14]. 

Sodium-selenite (Na_2_SeO_3_)-induced opacification of lens is widely used for studying the effects of various stresses on the lens, modelling various mechanisms of cataract formation and for screening potential anti-cataract agents [15]. Na_2_SeO_3_-overdose cataract is an extremely rapid and convenient model of nuclear cataract *in vivo* [16]. The mid-stage events producing the cataract include ROS generation, oxidative damage of the critical sulfhydryl groups of membrane Ca^2+^ATPase, calpain mediated proteolysis, precipitation of fragmented lens crystallins, and cytoskeletal proteins [17]. 

Various medicinal plants and their bioactive components, such as polyphenols and flavonoids, have been reported to render protective effects against Na_2_SeO_3_ cataract, by their antioxidant effect [18], [19].

Black tea (*Camellia sinensis*) is a widely popular beverage in Egypt and around the world. Tea leaves contain about 35% polyphenols by dry weight which exhibits a wide range of biological activities. Among these the catechins are converted to complex condensation products, namely theaflavins (TFs) and thearubigins (TGs), which impart the brew its rich characteristic [20]. 

Coffee (*Coffea arabica*) contains several compounds in particular phenols, niacin, magnesium, potassium and fiber [21]. Nevertheless, many other compounds with antioxidant properties [22] namely chlorogenic acid, flavonoids, melanoidins, furans, pyrroles and maltol have been found in coffee [23]. 

Hibiscus (*Rosa-Sinensis*) has anti-inflammatory, anti-tumor, wound healing, and laxative activities [24], [25]. However, there is little data related to the effect of hibiscus on cataract inhibition, specifically the anti-cataract properties. The present work aimed to study the potential antioxidative effects of consuming some common Egyptian beverages such as black instant coffee, black tea and hibiscus flowers in the development of lens opacification on sodium selenite-induced cataract in Sprague Dawley rats.

## Materials and methods

### Materials

Casein, vitamins, minerals and cellulose were obtained from El-Gomhoria Company, Cairo, Egypt. Other experimental diet ingredients such as corn starch, sucrose and soybean oil were obtained from the local market, Cairo, Egypt. Beverages include black teabags (El Arosa, El Fath Co., El Obour City, Egypt), Nescafe sachets (Misr Café Co., 6^th^ of October City, Egypt) and hibiscus teabags (Isis Co., Cairo, Egypt). Weaned male Sprague Dawley rats were purchased from the Animal House, Institute of Ophthalmology, Giza, Egypt. Dried packs of beverages were kept at room temperature (23–25°C) for a week until further use. 

### Chemicals

Unless otherwise stated, all chemicals were purchased Analar grade from Sigma Chemical Co., St. Louis, Missouri, USA. All chemicals were of the highest grade available. Folin-and Ciocalteu’s phenol reagent (Sigma-Aldrich, Buchs, Switzerland), sodium chloride 0.9% and gallic acid (3,4,5-trihydroxybenzoic acid), methanol, sodium carbonate and glutaraldehyde 25%. Na_2_SeO_3_ was obtained from Sigma Chemical Company (Merck’s Reagenzien, Darmstadt, Germany).

### Methods

#### Dry matter/moisture of beverages 

The amount of moisture and dry matter of samples were determined using an Infrared Moisture Determination Balance (FD-610-Kett Electric Laboratory, Tokyo, Japan) by weighing the 5 g sample at 80°C after 60 min (according to the manufacturer’s instructions). 

#### Determination of total phenols

Total phenol content was determined in the dried samples of black coffee, black tea and hibiscus by Folin-Ciocalteu reagent according to [26] with slight modification by [27]. The test is based on all phenolic compounds contained in the samples oxidized by Folin-Ciocalteu reagent. This reagent is formed from a mixture of phosphotungstic acid, H_3_PW_40_O_40_ and phosphomolybdic acid, H_3_PMo_12_O_40_, which, after oxidation of the phenols, is reduced to a mixture of blue oxides of tungsten W_8_O_23_, and molybdenum, Mo_8_O_23_. The blue coloration produced has a maximum absorption in the region of 750 nm, and is proportional to the total quantity of phenolic compounds originally present in the samples being studied.

#### Extraction of phenolics

Phenolic compounds were extracted by adding 1 g of ground (using a 6.4 cm diameter porcelain mortar and pestle) dried sample to 100 ml of aqueous methanol (1:1) and kept at room temperature (24–26°C) in a dark place for 24 h. The samples were centrifuged for 3 min. at 3000 rpm (Eppendorf Centrifuge 5804, Hamburg, Germany) and the supernatant was filtered using Whatman filter paper (Whatman, Maidstone, UK) into 10 mL test tube.

#### Gallic acid stock solution

In a 100 mL volumetric flask, 0.5 g of dry gallic acid (>99% purity) was dissolved in 10 mL methanol and diluted to volume with deionized water (DIW). To prepare a calibration curve, amounts of 0, 1, 2, 3, 5, and 10 mL of gallic acid stock solution were added into 100 mL volumetric flasks, and diluted to volume with DIW. These solutions had phenol concentrations of 0, 50, 100, 150, 250, and 500 mg/L gallic acid. The stability of gallic acid stock and standard solutions were tested (data not shown) and the loss was less than 3–5% for stocks and standard solutions stored over 4 weeks at 4°C, respectively. Since the test measures all phenolics, the choice of gallic acid as the standard is based on its availability as a stable and pure substance. 

#### Sodium carbonate solution

A 200 g sample of anhydrous sodium carbonate was dissolved in 800 mL of boiled and cooled (24 h) DIW. It was filtered using Whatman filter paper no 2 and DIW added up to the 1 L mark of the volumetric flask through the filter to dissolve the salt trapped in the filter paper. 

A 100 µL sample or DIW used as the blank was pipetted into separate tubes, and to each tube 1.6 mL DIW and 100 µL of the Folin-Ciocalteu reagent was added, and mixed well. The tubes were left for 8 min and then 300 µL of the sodium carbonate solution (20%) was added and each tube mixed well by several shakings by hand. The tubes were left at room temperature (~26–27°C) in a dark place for 2 h until the blue color developed and the absorbance of each solution measured at 750 nm using a 1 mL quartz cuvette (101-QS, Hellma GmbH, Mullheim, Germany) in an E-Chrom Tech Spectrophotometer (CT-2200, Taipei, Taiwan) against the blank (the “0 ml” solution). The absorbance vs. concentration of gallic acid standard curve was used to determine the gallic acid equivalence (GAE) in mg per 100 g dry weight (mg GAE/100 g).

#### Quality control

Known amounts of gallic acid were added to the samples before testing to obtain the percentage of total phenolics recovery. The recovery results with added gallic acid to the samples ranged from 95–106% for samples and 97% for the controls.

#### In vivo preliminary experiments 

A preliminary study was carried out in a separate group of weaned male Sprague Dawley rats to ascertain the required dose of sodium selenite (Na_2_SeO_3_) to induce cataract in rats with minimum toxicity to the liver. Weaned rats initially weighing 45±5 g (n=6) on day 20 were used for this study. Four doses of Na_2_SeO_3_ were trailed 5, 10 15, and 20 µmol/kg bodyweight/rat to optimize the best dose for rats. Therefore, two parameters were used to examine the histopathology of liver and eye lens: serum liver function (activities of glutamate oxaloacetate transaminase (GOT) and glutamate pyruvate transaminase (GPT). Upon the results (data not given), the effective dose of Na_2_SeO_3_ was fixed.

Before carrying out the main study, a beverage dose response study was conducted on 45±5 g on day 20 (n=6) by administering the beverages of instant black coffee, black tea and hibiscus tea at doses of 0.5, 0.75, 1.0 and 1.5 mg total phenols from starting material to rats injected with Na_2_SeO_3_-induced experimental cataract which was compared to the controls. The effective dose was fixed by determining the concentration of liver functions, TBARS were assayed in the experimental animals, which are common indicators of end products of lipid peroxidation [28]. A dose significant difference in activity was observed for the beverages indicating that the 0.75 mg total phenols obtained from beverages was non toxic (data not given). There was a non-significant increase in liver enzyme activity for the previous dose. Also a pathologist recommended the 0.75 dose for the healthy observation among this group compared to the rest of groups. From this study, 0.75 mg phenols and 15 µmol/kg bodyweight were fixed as the minimal effective dose for the production of maximum beneficial effects from beverages and Na_2_SeO_3_, respectively. 

#### Main in vivo experiment

Weaned male Sprague Dawley rats aged 20 days weighing 45±5 g were purchased from the Ophthalmology Institute Research, Giza, Egypt. The weaned rats were housed in an air-conditioned room at 22±2°C with a 12 h : 12 h light-dark cycle. Weaned rats were fed basal diet, Ain-93G diet [29] and water *ad libitum*. All procedures in this study complied with the ethical guidelines of the Animal Research Guidelines of the Ophthalmology Research Institute, Giza, Egypt. For the main study, 35 animals were grouped as follows with 7 rats in each group: the negative control group (NC) was fed Ain 93G and administered physiologic saline by stomach tube as a daily dose; the positive control group (PC) was fed Ain 93G and received a single dose of Na_2_SeO_3_ (15 µmol/kg bodyweight/rat); the black tea group (BTB) was fed Ain 93G, received Na_2_SeO_3_ and was administered 1 mL of tea as a daily dose; the instant black coffee group (IBC) was fed Ain 93G, received Na_2_SeO_3_ and was administered 1 mL of black instant coffee as a daily dose; the hibiscus group (HTB) was fed Ain 93G, received Na_2_SeO_3_ and was administered 1 mL of hibiscus as a daily dose. 

Injecting Na_2_SeO_3_ started on day 14 of the experiment for all rats mentioned above apart from the NC group (which did not receive Na_2_SeO_3_) and and the PC group (which was already injected with Na_2_SeO_3_ on day 1). Daily beverage administration started on day 1 of the experiment and was continued until day 30, when rats were sacrificed. 

#### Preparation of beverages for rats

Preparation of beverages were made up as a solution as for human consumption, 1 g of instant black coffee, black tea, or hibiscus was mixed with 900, 150, 188 mL of boiled tap water, to give 0.75 mg/mL total phenols for each group, respectively. The beverages were left until cooled down (~20°C) then a dose of 1 mL of each beverage was administered to its group. Amounts of total phenols in these beverages were calculated on the method described above for each beverage. 

#### Isolation of the lenses for histopathology

After treatment, weaned rats were sacrificed by means of an overdose of pentobarbital at a dose of 50 mg/kg body weight, given intraperitoneally, and the eyes were enucleated. Eye lens tissues were dissected out, washed in ice-cold saline to remove blood, and transferred to the pathology laboratory (Faculty of Veterinary, Cairo University).

#### Isolation of the lenses for biochemical analyses

The eyeballs were enucleated; tissues were soaked in 40 mL sodium chloride with 1 million units of penicillin [30]. Lenses were removed from the eye ball using micro-dissection according to [31]: materials required to isolate lenses were micro-dissecting scissors, curved, blunt tips (such as RS-5983, Roboz Surgical Instrument Co., Inc, Gaithersburg, MD); micro-dissecting tweezers, curved tip (such as Roboz #RS5137); suspension medium: Medium 199 (Trace Biosciences, Sydney, Australia) containing 0.1% bovine serum albumin, 100 units/ml penicillin, 100 µg/mL streptomycin, 2.5 µg/mL amphotericin. The rats were euthanized in accordance with the guidelines provided by the Institutes of Ophthalmology Giza, Egypt. The eyelids were removed with surgical scissors. The curved tweezers were used to press gently on the opposite sides of the eye socket to force the eye to bulge outward. A small incision was made on the posterior side of the eye with the scissors. 

By pressing with tweezers against the side of the eye opposite the incision, the lens and a small amount of attached vitreous body were forced out through the rupture, allowing the lens to be picked up with curved tweezers. Care has been taken not to break the lens capsule. The curved tweezers were used to transfer the lenses to a 60 mm plastic tissue culture dish containing 5 ml warm, sterile suspension medium. The lenses were prepared for biochemical analysis according to [30], and digested by 0.125% trypsin for 20 min. After sterile filtration with 220-mesh, bovine serum was used for termination of the digestion, and followed by centrifugation (1000 rpm/min) for 5 min. The suspension was collected by removing supernatant and repeated pipetting with 20% Dulbecco’s Modified Eagle’s medium (DMEM) culture medium. The supernatant obtained was stored at –70°C for a week in aliquots until use for the analysis of protein and enzyme activities. 

#### Biochemical analysis of lenses

At the end of the experiment, the rats were fasted overnight, anaesthetized and sacrificed to obtain blood samples. Each blood sample was placed in a dry clean centrifuge tube, and then centrifuged for 10 min at 2500 rpm to separate the serum. Serum was carefully separated into clean dry Wassermann tubes by using a Pasteur pipette and kept frozen for a month at –20°C until analyses. Malondialdehyde (MDA), reduced glutathione (GSH), superoxide dismutase (SOD), total nitric oxide (TNO), calcium ATPase, catalase, tumor necrosis factor-α, interleukin-1β (IL-1β) and total protein were carried out. 

#### Total proteins 

The protein content in the samples was determined by the method of [32], using bovine serum albumin as standard. For total protein estimation the lens homogenate was prepared in 5% trichloroacetic acid. The precipitated protein was dissolved in sodium hydroxide and used as aliquots for the estimation of total proteins. A soluble fraction of the protein was estimated by preparing the homogenates in double distilled water. The water-soluble supernatant was used for the estimation of soluble proteins.

#### Thiobarbituric reactive substances

TBARS were determined in the lenses by using OxiSelect™ TBARS Assay Kit (MDA quantitation) catalog number STA-330, (Cell Biolabs, Inc., San Diego, CA, USA). The TBARS assay kit is a tool for the direct quantitative measurement of MDA in the lenses. The principle of the TBARS test depends on the interaction between thiobarbituric acid (TBA) and malondialdhyde (MDA) incubated in water bath for 45 min at 95°C and acidified to pH 1 with hydrochloric acid 6M, the resultant pink color is measured at 532 nm by a spectrophotometer [28]. The difference in absorbance was calculated and TBARS concentrations were determined from a standard curve. 

#### Total glutathione (reduced) content

Total GSH content was measured as described by [33]. The reaction mixture containing 1.2 mL EDTA (0.02 M), 1 mL DIW, 250 µl 50% trichloroacetic acid, 50 µL Tris buffer (0.4 M, pH 8.9) was centrifuged at 300 × g for 15 min. Clear supernatant (500 µL) was mixed with 1 mL of 0.4 M Tris buffer (containing 0.02 M EDTA, pH 8.9), 100 µl of 0.01 M DTNB (5,5-dithio-bis-(2-nitrobenzoic acid) and 100 µL cell extract. The mixture was incubated at 37°C for 25 min and the yellow colour developing was read at 412 nm against blank. The enzyme activity was calculated taking the extinction coefficient as 14,150 L M^–^1cm^–^1. 

#### Superoxide dismutase

Tissue SOD was determined quantitatively *in vitro* by using enzyme-linked immunosorbent assay (ELISA) (Kamiya Biomedical Co., WA, USA, kit cat. No. KT-60703) according to the method of [34]. The assay is based on the conversion of nitroblue tetrazolium (NBT) to NBT-diformazan by superoxide ions generated from the reaction of xanthine and oxygen catalyzed by xanthine oxidase. SOD reduces superoxide ion concentration, and hinders the appearance of NBT-diformazan. The absorbance was measured at 550 nm. SOD activity in samples was determined from a standard curve and expressed as units/mg lens protein.

#### Total nitric oxide

According to the method of [35], the total nitric oxide detection kit (Enzo Life Science, NY, USA) was used. The assay was based on the enzymatic conversion of nitrate to nitrite by the enzyme nitrate reductase, followed by the Griess reaction to form a colored azo dye product. Quantification is performed by measuring absorption at 550 nm. Only 0.050 mL diluted sample needed per well saves precious sample. Briefly, 200 µl reaction buffer were added to the blank tube (duplicate), and 50 µL sample, 25 µL NADH, and 25 µL nitrate reductase to the sample tube (duplicate). The tubes were incubated for 30 min at 37°C. Thereafter, 50 µL Griess I and 50 µL Griess II were added to the sample tubes, stirred, and left to stand at room temperature for 10 min before their absorption was measured at 540 nm against the blank. A standard curve was used to calculate the concentration in Pg/mg lens. The data was linearized by plotting the log of the NO concentrations versus the log of the OD and the best fit line was determined by regression analysis. 

#### Calcium-ATPase

Ca-ATPase activity was measured by Canine (MyBioSource, CA, USA) Ca (2+)-ATPase ELISA kit, catalog number: MBS755220. Ca (2+)-ATPase ELISA kit applies the competitive enzyme immunoassay technique utilizing a monoclonal anti-Ca(2+)-ATPase antibody and Ca (2+)-ATPase-horseradish peroxidase (HRP) conjugate. The assay sample and buffer were incubated together with Ca (2+)-ATPase-HRP conjugate in pre-coated plate for 1 h. After the incubation period, the wells were decanted and washed five times. The wells are then incubated with a substrate for HRP enzyme. The product of the enzyme-substrate reaction forms a blue coloured complex. Finally, a stop solution is added to stop the reaction, which will then turn the solution yellow. The intensity of colour is measured spectrophotometrically at 450 nm in a microplate reader (model 3550; Bio-Rad, Hercules CA, USA).

#### Catalase

Catalase was determined quantitatively in the lenses of the rats by catalase kit Cat. no. KT-711, (Kamiya Biomedical Co., WA, USA). Bovine catalase calibrator is provided to generate a calibration curve for the assay and all samples read off of the calibration curve. Samples were diluted in the provided assay buffer and added to the wells of a half area clear plate. Hydrogen peroxide was added to each well and the plate incubated at room temperature for 30 min. The supplied colorimetric detection reagent was added, followed by diluted HRP and incubated at room temperature for 15 min. The HRP reacts with the substrate in the presence of hydrogen peroxide to convert the colorless substrate into a pink-colored product. The colored product was read at 560 nm. Increasing levels of catalase in the samples causes decrease in H_2_O_2_ concentration and a reduction in pink product. The activity of the catalase in the sample is calculated after making a suitable correction for any dilution, the results were expressed in terms of µmol/g lens protein. 

#### Tumor necrosis factor alpha 

Tumor necrosis factor alpha (TNF-α) was determined quantitatively in the lenses of the rats by assay kit (Immuno-Biological Laboratories Co., Ltd., Guma, Japan) code No. 271945. The kit is a solid phase sandwich ELISA using 2 types of high specific antibodies. Tetra methyl benzidine (TMB) is used as coloring agent (chromogen). The strength of coloring is proportional to the quantities of rat TNF-α. The plate was read at 450 nm against a reagent blank within 30 min after addition of stop solution.

#### Determination of interleukin-1β 

Interleukin-1β was determined quantitatively in the lenses of the rats by the assay kit (Immuno-Biological Laboratories Co., Ltd., Guma, Japan) Code No. 27193. An anti-Rat IL-1β coating antibody is adsorbed onto microwells. Rat IL-1β present in the sample or standard binds to antibodies adsorbed to the microwells. A biotin-conjugated anti-Rat IL-1β antibody is added and binds to Rat IL-1β captured by the first antibody. Following incubation unbound biotin-conjugated anti-Rat IL-1β antibody is removed during a wash step. Streptavidin-HRP is added and binds to the biotin-conjugated anti-Rat IL-1β antibody. Following incubation unbound Streptavidin-HRP is removed during a wash step, and substrate solution reactive with HRP is added to the wells. A colored product is formed in proportion to the amount of Rat IL-1β present in the sample or standard. The reaction is terminated by addition of acid and absorbance is measured at 450 nm. A standard curve is prepared from Rat IL-1β standard dilutions and Rat IL-1β sample concentration determined. 

#### Histological examination

Lenses were dissected from the globe and fixed in 2.5% glutaraldehyde, processed by conventional method and sectioned at 4–5 µm. The slides were stained by hematoxyline and eosin stain and examined by light microscope [36].

#### Statistical analysis

Standard deviation (STD) and coefficient of variation (CV) were calculated using Excel 2003 Microsoft Windows Operating System, Windows 7 Home Premium, for the phenol content and biochemical analyses. Statistical analysis was performed by one-way analysis of variance (ANOVA) followed by Dunncan-Test, using the statistical package for social science (SPSS) version 16 to compare all treated groups. Values were expressed as mean ± SEM and P<0.05 was considered to be significant [37].

## Results

### In vitro experiment

The dry matter accounted for approximately 99.6, 99.4 and 99.6% in black tea, black instant coffee, and hibiscus respectively. The total phenols in the instant black coffee, black tea, and hibiscus were 210, 40, and 44 mg/g dry weight gallic acid equivalent, respectively. 

### In vivo experiment

The results in Table 1 (Tab. 1) and Table 2 (Tab. 2) show the effects of the consumption of common beverages on GSH, MDA, CAT, SOD, TNO, Ca ATPase, IL-1β, TNF-α, total protein, soluble protein and insoluble protein levels in rats with sodium selenite-induced cataract. There were decreased levels (statistically significant P<0.05) of malondialdehyde, total nitric oxide, Ca ATPase, tumor necrosis factor-α, interleukin-1β, superoxide dismutase in the lenses of the coffee group compared to PC. Conversely, we found increased levels (statistically significant P<0.05) of total protein, reduced glutathione, catalase in the lenses of the coffee group compared to PC. The hierarchical potency of beverages was coffee > hibiscus > tea. The coffee group gave the best results compared to the hibiscus and black tea rat groups. 

### Histopathology

**Figure 1:**
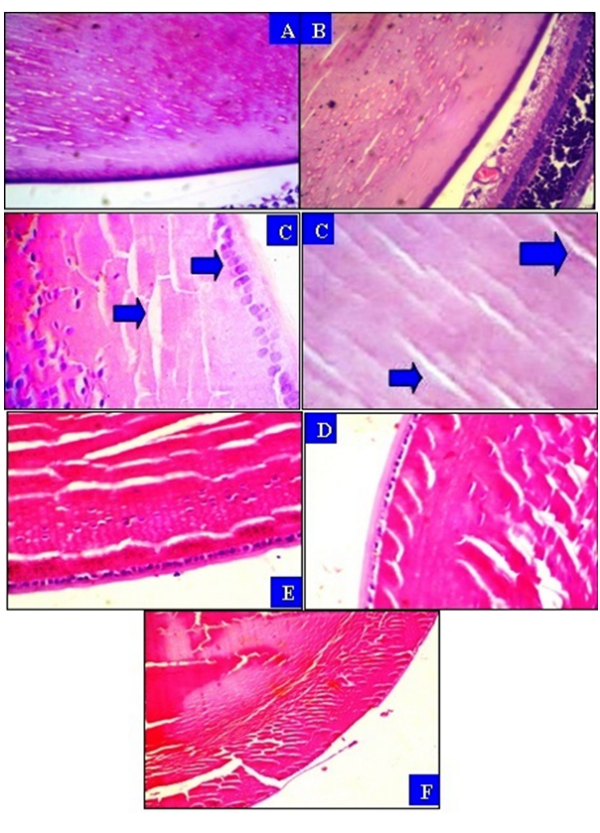
(A) Lens of a rat from the NC group showing vacuolation and granulation of lens fibre which is artifact due to fixation (H & E x 400). (B) Eye tissue of a rat from the coffee group showing a natural gap between the lens and the retina in the eye (H & E x 400). (C) Lens of a rat from the PC group showing hyperplasia of the lens epithelium (left) and a wide gaping among the lens fibers (right). (D) Lens of a rat from the black tea group showing a normal capsule. (E) Lens of the group treated with hibiscus showing a line of nuclei visible due to the orientation of the section (H & E x 400). (F) Lens of a rat from the hibiscus group showing artificial absence (H & E x 100).

**Figure 2:**
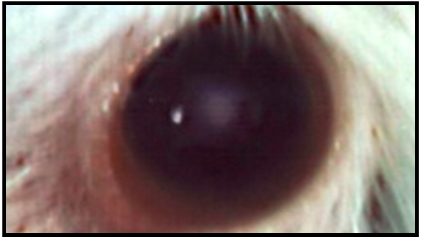
Cataract formation in the PC group 30 days after injection of Na_2_SeO_3_

The histological changes in the lenses of rat groups injected with Na_2_SeO_3_ were characterized by mild to moderate histopathological changes (Figure 1 (Fig. 1)). Administration of a single dose of 15 µmol/kg Na_2_SeO_3_ resulted in appearance of opacity after 1 week, and this opacification was found to progressively increase with time after 30 days (Figure 2 (Fig. 2)). The results presented in Figure 1 (Fig. 1) indicate that treatment of rats with standardized dose from black tea, black coffee and hibiscus tea once daily, starting at the day 1 to day 30 with a single dose of Na_2_SeO_3_, delayed the cataract formation compared to the PC group. The developed opacity with respect to the total number of lenses in the PC group was 5 rats with cataract out of 7 rats. No cataract has been observed in the treated groups compared to the control group. In the treated rat groups no histopathological changes were found (Figure 1 (Fig. 1)).

## Discussion

The current study aimed to investigate the effects of daily consumption of common beverages such as black instant coffee, hibiscus teabags and black tea on the development of lens opacification mediated sodium selenite-induced cataract in Sprague Dawley rats. A series of chemical, biochemical and histological studies were done in the lenses of rats to evaluate the anticataractogenic properties of the daily consumption of common beverages. The beverages were served to rats in their original form (as they are served to humans) without extraction and then tested in an *in vivo* model of cataractogenesis. The rats were treated with daily standardized doses from the beverages being studied. Biochemical analyses were done on the lenses of the rat groups such as malondialdehyde (MDA), reduced glutathione (GSH), catalase (CAT), superoxide dismutase (SOD), total nitric oxide (TNO), Ca ATPase, interleukin-1β (IL-1β), tumor necrosis factor-α (TNF-α) and total protein (TP) (soluble and insoluble).

Our results show the superior effect of the black instant coffee on the biochemical parameters being studied, although we gave a standardized dose of phenols from the three beverages. It remained unclear why coffee shows the best results. This would mean that there are other substances occurring in the instant black coffee that promoted coffee to be the most effective beverage to delay the cataract formation significantly (P<0.05) in the weaned rats compared to the rest of groups. Clearly hibiscus and black tea do not have enough amounts of these substances. A significant increase in enzyme antioxidants in the lenses when rats shifted to instant black coffee would mean that these co-phenols are capable of protecting these antioxidants from oxidation by Na_2_SeO_3_ in the lenses. 

Our results provide several lines that are highly relevant to our overall efforts in designing strategies for delaying or preventing cataractogenesis through the daily consumption of common beverages: 1) the reactive end product of lipid peroxidation MDA may cause opacification of lenses in agreement with [38], [39]; and 2) cataractogenic effect of sodium selenite is significantly affecting enzyme antioxidants, inflammatory markers and protein content. The administration of instant black coffee ranked as best treatment. The preventive effect of coffee may correlate to alkaloids in black instant coffee. Coffee beans contain two types of alkaloids, caffeine and trigonelline, as major components [40]. The studies on possible beneficial and therapeutic effects of caffeine against the ageing eye diseases have been very limited except some recent preliminary reports suggesting that it has a potential to slow down cataract progression. A study found the micromolar amounts of topical caffeine (eye drops) significantly effective in inhibiting the formation of galactose cataract, strongly suggesting its possible usefulness against diabetic cataracts. The effects are attributed to its ability to prevent oxidative stress and the consequent maintenance of metabolic and transport functions in tissues, in addition to preventing the induction of apoptosis [41]. 

Another study based on *in vitro* organ culture experiments showing its effectiveness in offering protection against lens damage caused by UV exposure, as reflected by the severe inhibition of the active cation transport process as well as the depletion of GSH. Incorporation of caffeine in the culture medium protects against the induction of these deleterious effects [42], [43]. It also prevents the loss of tissue transparency associated with UV exposure. Such a loss of transparency *in vivo* would directly interfere with transmission and refraction of light coming into the eye and its focusing on the retina. 

Interestingly in a preliminary study, caffeine has also been shown to prevent phototoxic damage to retina [44]. The anticataractogenic potential of the compound *in vivo* is suggestible also by preliminary studies showing that formation of cataracts in rats maintained on a high galactose (24%) diet is significantly inhibited if the galactose diet is fortified with 1% caffeine [43]. 

### The scenario of the study behind the hypothesis

In the present study, the authors hypothesized that if these beverages are rich in phenolics and consumed in early stage of life (childhood), this may lead to protection from cataract, therefore we set the script of this study on the weaned rats. 

The weaned rats were administered the standardized dose of beverages for 14 days as a prophylactic strategy before injecting them with Na_2_SeO_3_. However, on the day 14 all rats were injected once with Na_2_SeO_3_ (except NC group). One mL of coffee, hibiscus and tea were administered orally to the rat groups as a daily dose which supplies 0.75 mg phenols.

Interestingly, Vinson and Zhang [45] used an animal study to test standard green or black tea as prepared for human consumption. They had a control group of animals given normal chow and water. The diabetic groups were rendered diabetic with streptozotocin and divided into groups: diabetic control, diabetic green tea and diabetic black tea. All animals were given rat chow and artificial sweetener in the drinking water or tea (made up as a 1.25% solution as for human consumption). Both tea treatments significantly decreased the severity of cataracts and hyperglycaemia in plasma and lens. Both teas significantly decreased plasma protein glycation, red blood cell sorbitol and plasma lipid peroxidation in the lens. 

The present study showed that enzyme antioxidants and lens protein were preserved significantly (P<0.05) in the coffee group compared to PC. This may explain the important role of antioxidants enzymes in protecting lenses from the deleterious effects of lipid peroxidation. The apparent correlation between the protective effect of coffee, hibiscus and tea against sodium selenite-mediated cataractogenesis in our model was statistically significant at P<0.05.

In our study, the period from the Na_2_SeO_3_ injection until the end of the study was 16 days only what may be classified as the first stage of cortical cataract according to [45]. Our results are in line with Anderson and coworkers [46] who showed that sodium selenite caused wrinkling of the lens capsule, especially in the posterior part, indicating loss of material from the lens. Also no deep cortical opacity was observed with the slit lamp, but histological examination showed that pathological changes were present in the entire cortex. 

The literature in this area of how the common beverages may delay the formation of cataract is rare, but the results of our study seem promising for a clinical trial. 

## Notes

### Competing interests

The authors declare that they have no competing interests.

### Acknowledgment

This work is totally supported financially by Enas El Okda, Women’s College, Ain Shams University, Cairo, Egypt. We are gratefully acknowledging the Department of Nutrition and Food Science, Faculty of Home Economics, Helwan University for letting us work in the Food Science Laboratories to prepare beverage samples and phenol assay. We are also grateful to Mr. Saeed Hassanien the Institute of Ophthalmology Research for his constant technical assistance in the *in vivo* experiment. Also we would like to thank the Ideal Solution Academy Laboratory (ISA) Cairo, Egypt for the biochemical analyses in serum and lenses. Also we are acknowledging the great job of examining tissue samples from the Department of Pathology, Faculty of Veterinary, Cairo University. We finally thank Prof. Manal K Abdel-Rahman for her revising the manuscript in spite of her limited time and for her guidance.

## Figures and Tables

**Table 1:**
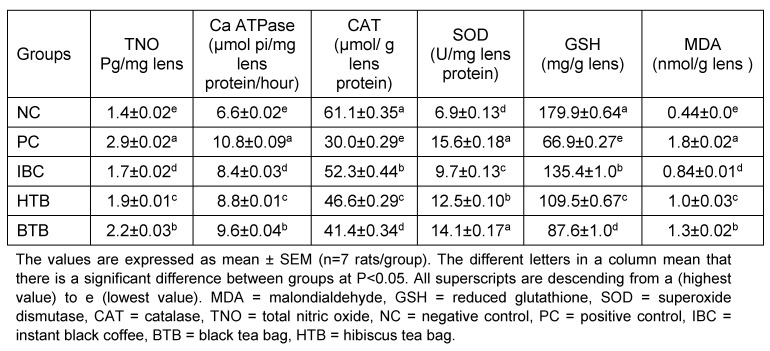
Effects of black instant coffee, hibiscus and black tea beverages on the GSH, MDA, CAT, SOD, TNO, and Ca-ATPase levels of the lenses in rats with sodium selenite-induced cataract

**Table 2:**
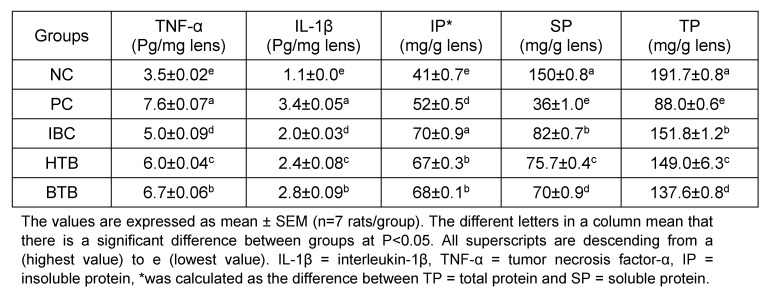
Effects of black instant coffee, hibiscus and black tea beverages on the IL-1β, TNF-α, total protein, soluble protein, and insoluble protein levels of the lenses in rats with sodium selenite-induced cataract
